# Healthcare Indicators in Lithuania: A Descriptive Analysis of Their Contextual Relevance to Health Literacy

**DOI:** 10.3390/clinpract16070134

**Published:** 2026-07-17

**Authors:** Sonata Čerkauskaitė, Alina Liepinaitienė

**Affiliations:** Healthcare Research Center, SMK College of Applied Sciences, Vilties St. 2, LT-46326 Kaunas, Lithuania; alina.liepinaitiene@smk.lt

**Keywords:** health literacy, healthcare, cardiovascular diseases

## Abstract

**Background/Objectives**: Chronic non-communicable diseases remain one of the main public health problems. Increasing multimorbidity and the importance of health literacy (HL) emphasize the need for a comprehensive assessment of health indicators. The aim of this study was to assess the main health indicators of the Lithuanian population and trends in the use of healthcare services and to discuss the relevance of these indicators in the context of HL, based on the links between HL and these indicators described in the scientific literature. **Methods**: A retrospective longitudinal descriptive study was performed using publicly available Lithuanian population health statistics of 2005–2024. Mortality, morbidity, avoidable hospitalizations, subjective health assessment, and utilization of healthcare services and preventive programs were analyzed using descriptive statistical analysis. HL was not directly measured but was used as a conceptual framework for interpreting the findings. **Results**: In Lithuania, the highest mortality rate is due to cardiovascular diseases (CVDs) (~50.8%). CVD and infectious diseases also dominate the structure of avoidable hospitalizations, and their rates vary greatly across municipalities, being higher in less urbanized areas. The assessment of the population’s health is improving over time, but gender differences remain in the use of healthcare services and preventive programs. **Conclusions**: The findings demonstrate a high burden of chronic diseases and regional disparities in healthcare utilization in Lithuania. HL may provide a useful context for interpreting these findings, although it was not directly assessed. Future studies should directly evaluate HL and its association with health indicators in the Lithuanian population.

## 1. Introduction

In Lithuania, chronic non-communicable diseases (NCDs) remain one of the largest public health problems and account for the largest share of morbidity, mortality and burden on the healthcare system. Cardiovascular diseases (CVDs) remain the leading cause of death, accounting for about half of all deaths in the country, and Lithuania continues to be among the countries in the European Union (EU) with one of the highest CVD mortality rates. In addition, the increasing prevalence of NCDs, increasing multimorbidity and the scale of avoidable hospitalizations indicate a significant impact of these diseases on the health of the population and the healthcare system. These trends are further strengthened by demographic changes, such as the aging of the population and the decreasing number of the working-age population, which increase the prevalence of NCDs and the need for long-term healthcare services [1,2,3].

Studies show that the burden of NCDs is not only about clinical health outcomes, but also about patients’ quality of life, functional status, and health system performance. The increasing prevalence of NCDs, including multimorbidity, is associated with increased readmissions, longer length of stay, higher healthcare costs, and higher mortality risk [4,5]. These indicators are important for assessing health system performance and planning healthcare resources. In today’s healthcare system, increasing attention is being paid to health literacy (HL), which is the ability of patients to find, understand, evaluate, and apply health-related information to make health-related decisions. Studies show that poor HL is associated with poorer chronic disease management, lower adherence to treatment recommendations, lower participation in preventive programs, and higher risk of hospitalization [6].

HL also encompasses not only the ability to understand health-related information, but also to critically evaluate it, communicate effectively with health professionals, and make informed decisions about one’s health. These skills are considered important for the effective management of NCDs, active participation in prevention programs, and appropriate use of health services [7,8].

Recent evidence also indicates that limited HL remains a significant public health issue in Lithuania. A population-based study conducted in Lithuania found that approximately 73% of adults have inadequate or problematic HL, highlighting the need to better understand health indicators that may be relevant in this context [9].

In this context, health indicators such as mortality, morbidity, avoidable hospitalizations, subjective health assessment, and health service utilization can be considered important in the context of HL. In this regard, this article aims to assess the main health indicators of the Lithuanian population and trends in the use of healthcare services and to discuss the relevance of these indicators in the context of HL, based on the links between HL and these indicators described in the scientific literature.

This study complements existing Lithuanian public health research by systematically describing key health indicators and trends in healthcare service utilization and discussing their relevance in the context of HL, based on data from previous research.

## 2. Materials and Methods

### 2.1. Study Design and Data Framework

A retrospective longitudinal descriptive study was conducted based on secondary analysis of publicly available statistical data on the health of the Lithuanian population. The main data sources are the Lithuanian Institute of Hygiene Health statistics [10] and the Lithuanian Official Statistics Portal [11]. Data from the Lithuanian Institute of Hygiene’s Report on Avoidable Hospitalization Rates in Lithuania (2024) [12] were also used.

The study indicators were selected based on the World Health Organization’s Health System Performance Assessment Framework, which recommends population health outcomes, health service utilization, and coverage of preventive measures as key indicators for evaluating health system performance [13]. These indicators were chosen in this study not as direct measures of HL, but as indicators of health system and population health, the links between which and HL are widely discussed in the scientific literature [14,15]. Therefore, they are considered appropriate to discuss in the context HL. HL was not analyzed as an explanatory variable in this study. It was used as a conceptual framework for interpreting selected health indicators based on data from previous research.

In view of this, the following groups of indicators were selected for analysis: registered prevalence of CVD and type 2 diabetes (number of registered cases of 1000 population), avoidable hospitalizations, coverage of preventive programs and influenza vaccination. Chronic respiratory diseases were not included in the detailed morbidity analysis, in order to limit the study to those chronic diseases whose management, based on previous studies, is most closely related to HL and patient self-care [16]. The main analysis was focused on the adult population (18 years and older) in order to reflect indicators of chronic non-communicable disease management. Taking into account the specifics of individual indicators, the analysis of cancer screening was performed only for the target age groups defined in national preventive programs, and the analysis of influenza vaccination was limited to the population aged 65 years and older. Data from children (under 18 years) were not included in the analysis.

### 2.2. Data Variables

The following groups of research indicators were selected for the analysis (Table 1): (1) Mortality structure by main causes of death (%). (2) Morbidity indicators, including CVD and type 2 diabetes prevalence (number of cases per 1000 population). CVDs were defined according to ICD-10-AM codes I00–I99, while type 2 diabetes mellitus was identified using ICD-10-AM code E11. (3) Avoidable hospitalizations, expressed as number of cases per 1000 population. (4) Subjective health assessment data were obtained from the European Health Interview Survey (EHIS), as reported by the Official Statistics Portal of Lithuania. EHIS is a nationally representative population survey conducted using a standardized methodology across European countries. Self-rated health was assessed using the standard five-category question (“very good”, “good”, “fair”, “bad”, “very bad”). (5) Use of healthcare services, including visits to family doctors and specialist doctors (%) (in the last 12 months). (6) Participation in preventive programs, including oncological disease screenings and influenza vaccination (%).

Avoidable hospitalizations are defined as hospitalizations due to outpatient-managed diseases or conditions that could have been avoided through effective primary healthcare, preventive measures and timely initiation of treatment. The indicators were calculated based on the national methodology approved by the Order of the Minister of Health of the Republic of Lithuania, using data from the Compulsory Health Insurance Information System (SVEIDRA). The analysis included only cases of active treatment hospitalizations, selected according to the main diagnosis (ICD-10-AM codes), excluding day hospital and day surgery cases.

For the purpose of regional comparison, municipalities were categorized according to their level of urbanization, taking into account population density and the complexity of the available healthcare infrastructure. This categorization was applied to reflect differences in healthcare accessibility and the availability of specialized healthcare services between Lithuania’s largest urban municipalities and the remaining municipalities, which may influence healthcare service utilization and selected health indicators. ‘More urbanized areas’ were defined as the five major city municipalities (Vilnius, Kaunas, Klaipėda, Šiauliai, and Panevėžys), which are characterized by higher population density and the presence of university or republic-level tertiary hospitals. ‘Less urbanized or peripheral areas’ were defined as the remaining district municipalities, where healthcare services are primarily delivered through primary care centers and secondary-level district hospitals.

### 2.3. Statistical Analysis

Descriptive statistical analysis was performed. The indicators are presented as follows: structural indicators in percentages (%); morbidity and hospitalization indicators in number of cases per 1000 population. The analyzed periods covered 2005–2024 (depending on the availability of the indicator). The data were analyzed in the general population and, where possible, stratified by gender. Changes in the indicators were assessed descriptively by visual inspection of the data. No formal statistical trend analysis was performed.

This study used only publicly available summarized secondary statistical data; therefore, ethics committee approval was not required. Data were aggregated and anonymized at the source.

## 3. Results

The study results provide information on mortality, morbidity trends, patterns of avoidable hospitalizations, subjective health assessment, and utilization of healthcare services and preventive programs in Lithuania. The findings are generalized to the general population and, where applicable, analyzed by gender and over time.

**Figure 1:**
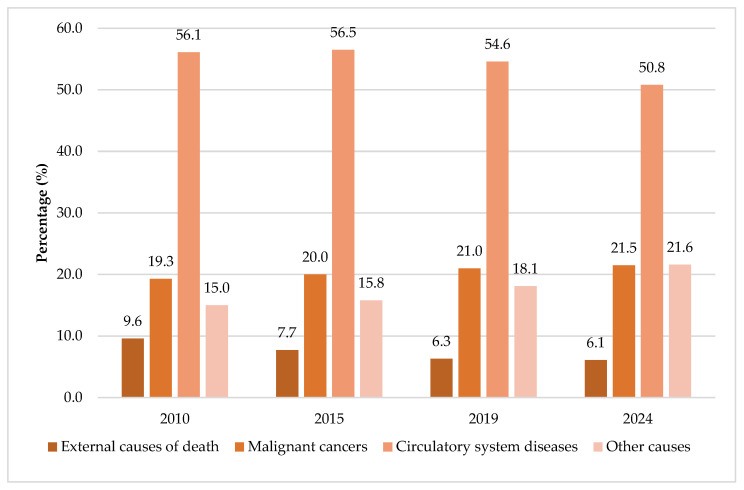
Changes in the structure of the main causes of death in Lithuania, 2010–2024 [10].

The structure of the causes of death of the Lithuanian population has remained unchanged for many years. The three main causes of death dominate—diseases of the circulatory system, malignant tumors and external causes of death. In all the years analyzed, diseases of the circulatory system accounted for the largest share of all deaths, although their share gradually decreased—from 56.1% in 2010 to 50.8% in 2024. Meanwhile, the share of deaths due to malignant tumors consistently increased—from 19.3% to 21.5%. The share of external causes (including injuries, accidents, suicides, and other external events) of death decreased from 9.6% to 6.1% during the analyzed period. Despite these changes, the distribution of the main causes of death remains similar (Figure 1).

**Figure 2:**
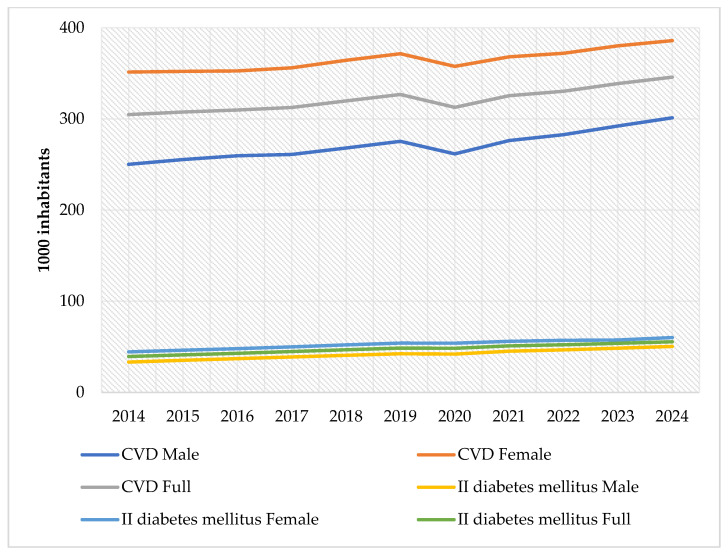
Trends in the registered prevalence of cardiovascular diseases and type 2 diabetes mellitus per 1000 population in Lithuania [10].

During the period of 2014–2024, a consistent upward trend in the registered prevalence of CVD was observed both in the general population and in the groups of men and women. A temporary decrease in 2020 did not change the overall growth trend. The overall registered prevalence of CVD increased from 304.78 cases per 1000 population in 2014 to 345.77 cases in 2024. A similar upward trend is recorded among women and men in cases per 1000 population. In all analyzed years, the registered prevalence of CVD in women remains higher than in men, and the overall population rate (Figure 2).

During the analyzed period, a consistent upward trend in the registered prevalence of type 2 diabetes mellitus in all groups was observed. The overall registered prevalence of rate increased from 39.27 cases per 1000 population in 2014 to 55.56 cases in 2024. In all analyzed years, the registered prevalence of type 2 diabetes mellitus in women was higher than in men. A slight decrease in registered prevalence was recorded in 2020 (this was likely due to the COVID-19 pandemic and reduced access to diagnostics, rather than a true drop in disease prevalence), when the indicators decreased in all groups, but in subsequent years, consistent growth was again observed (Figure 2).

**Figure 3:**
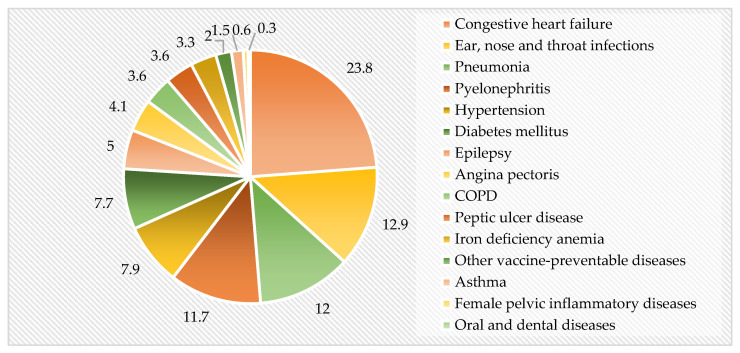
Structure of avoidable causes of hospitalizations in Lithuania in 2023, in percentages [12].

In 2023, the largest proportion of avoidable hospitalizations in Lithuania was attributable to congestive heart failure (23.8%), followed by ear, nose and throat infections (12.9%), pneumonia (12.0%), pyelonephritis (11.7%), hypertension (7.9%), and diabetes mellitus (7.7%). The remaining conditions accounted for smaller proportions of avoidable hospitalizations (Figure 3).

**Figure 4:**
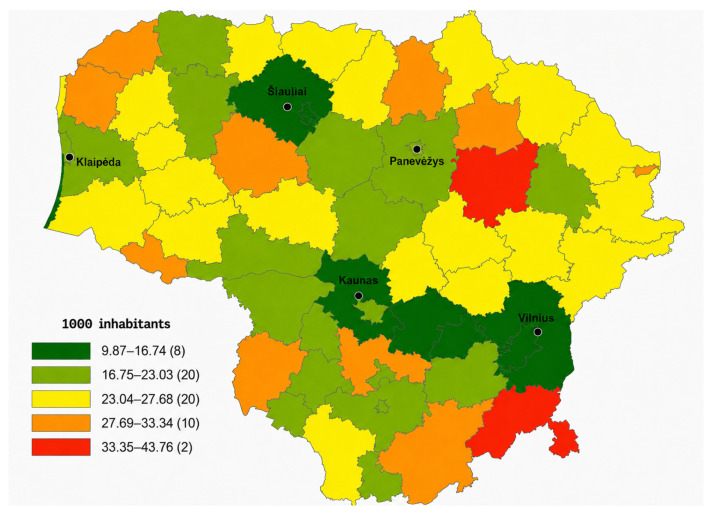
Prevalence of avoidable hospitalization in Lithuanian municipalities in 2023 [12].

In 2023, avoidable hospitalization rates in Lithuanian municipalities showed significant regional variation (Figure 4). Rates ranged from fewer than 10 to more than 40 cases per 1000 inhabitants. Lower rates were more common in the five largest urban municipalities, while higher rates were observed in most other municipalities. These differences indicate an uneven distribution of avoidable hospitalizations across Lithuanian regions and may reflect differences in the availability of healthcare services, their organization, and other factors related to the functioning of the health system. Given the links described in previous studies, these rates can also be discussed in the context of HL, but HL was not directly assessed in this study.

**Figure 5:**
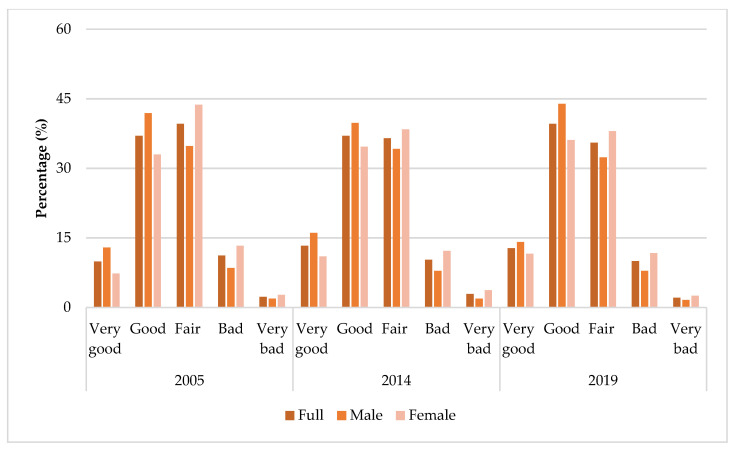
Lithuanian residents’ assessment of their own health [11].

When analyzing the subjective health assessment of the Lithuanian population by gender in the long term (2005, 2014, 2019), the trend established in the proportion of the population who assess their health positively is increasing. The growth of the category “good” is particularly noticeable, which in 2019 reaches its highest values both in the general population and in both gender groups. When analyzing gender differences, a consistent pattern is visible: men more often than women assess their health positively (“very good” and “good”), while women more often tend to choose a fair or negative assessment. It is important to note that the category “fair” makes up one of the largest shares in all periods, although its significance is slightly decreasing, indicating a shift towards a better assessment of well-being. Meanwhile, the assessment of “very bad” health remains rare and almost unchanged (Figure 5).

Data on the use of healthcare services and participation in preventive programs by the population show that the use of family doctors’ services remains relatively stable during the analyzed period, but significant gender differences are observed. In 2014, 74.4% of the total population visited family doctors, of which 66.5% were men and 80.9% were women. In 2019, the overall indicator decreased slightly to 73.6%, the share of men remained essentially unchanged (66.4%), and the share of women decreased to 79.6%. A similar trend is observed when assessing visits to specialist doctors (Table 2).

Influenza vaccination of the elderly population (65 years and older) shows slight fluctuations. In 2022, 22.5% of persons in this age group were vaccinated, in 2023 the indicator increased to 24.1%, but in 2024 it decreased again to 22.4%. This suggests that vaccination coverage remains relatively low and unstable. The indicators of preventive programs reveal different trends depending on the type of program. Participation in the breast cancer prevention program (mammography) is consistently increasing: from 57.6% in 2022 to 59.7% in 2023 and 61.2% in 2024. In cervical cancer prevention, 60.3% of the target group participated in 2023, and in 2024 this indicator increased significantly to 74.0%. Meanwhile, the indicators of non-participation in the colorectal cancer prevention program show the opposite trend. In 2014, the non-participation rate reached 86.4% in the 50–59 age group, 79.3% in the 60–69 age group and 77.1% in the 70–74 age group. In 2019 these rates decreased to 73.9%, 63.3% and 60.8%, respectively, indicating growing engagement in this preventive program across all age groups (Table 3).

## 4. Discussion

The results of this study confirm that chronic non-communicable diseases, especially cardiovascular diseases (CVDs), remain a dominant health problem in Lithuania, accounting for the largest share of mortality. This finding is consistent with previous reports showing that Lithuania continues to have one of the highest cardiovascular mortality rates among EU countries, despite the overall decline in cardiovascular mortality observed across the EU [2,3]. More than half of all deaths were found to be related to these diseases, which is in line with international trends showing that CVDs remain the leading cause of death in many European countries [17]. This may be related not only to the aging population and lifestyle risk factors, such as smoking, lack of physical activity, unbalanced diet, obesity or alcohol consumption [18], but also to insufficient disease prevention, insufficient management of chronic diseases and limited patient involvement in their healthcare. Research shows that lower HL is associated with poorer control of cardiovascular risk factors, lower adherence to treatment recommendations, and worse disease outcomes [19,20]. It has also been found that insufficient HL hinders patients’ ability to recognize symptoms, understand medical information, and effectively use the healthcare system, and therefore may be associated with delayed diagnosis and poorer disease prognosis [8,21].

The observed steady increase in the registered prevalence of cardiovascular diseases and type 2 diabetes indicates an increasing burden of chronic diseases on the health system. These trends can be explained not only by epidemiological factors, but also by better disease diagnostics and earlier detection [22]. In addition, the observed increase in registered prevalence may reflect several concurrent processes, including a true increase in the burden of chronic diseases associated with population aging and lifestyle-related risk factors, improved case detection through wider access to diagnostic services and preventive programs, and changes in disease registration and reporting practices. Therefore, the observed patterns should be interpreted with caution, as they may reflect both changes in population health and improvements in the identification and recording of chronic diseases.

However, it is important to note that the higher registered prevalence, especially among women, may also be related to their more frequent contact with the healthcare system and higher use of healthcare services. Studies show that women use preventive services more often than men, regularly attend preventive check-ups, and consult doctors more often, which is why they are diagnosed with diseases more often [23]. Furthermore, a temporary slowdown in the increase in registered prevalence observed during the COVID-19 pandemic may be related to disruptions in healthcare utilization and reduced opportunities for chronic disease diagnosis rather than to a true reduction in disease occurrence. Similar effects have been reported in other countries following the COVID-19 pandemic [24].

The analysis of the structure of avoidable hospitalizations showed that a large proportion of them are conditions that could be effectively managed in outpatient settings. This indicates the importance of strengthening primary healthcare. Visible territorial differences suggest that the availability and organization of healthcare services are not the same in different regions of Lithuania. The higher number of hospitalizations in less urbanized municipalities may be related to various interrelated factors, including more limited access to healthcare services, uneven distribution of healthcare professionals, socio-economic differences and other factors. Based on previous research, one of the possible explanations for these differences may be differences in HL, but in this study, HL was not directly assessed; therefore, such a link can only be discussed as an interpretative assumption.

These results suggest that the prevalence of avoidable hospitalizations may be related not only to the accessibility of healthcare services, but also to the ability of the population to understand health information and appropriately manage chronic diseases. The scientific literature consistently emphasizes that lower HL is associated with later seeking medical care, poorer adherence to medication regimens, lower participation in preventive programs, and a higher risk of hospitalizations [19,25].

Improving subjective health assessments may indicate positive public health trends, but persistent gender differences suggest that health perceptions are related not only to objective health status, but also to social, psychological and behavioral factors. Research shows that higher HL is significantly associated with better self-assessment of one’s health, healthier lifestyles and more active participation in health-promoting activities [26,27]. Individuals with higher HL tend to better understand health information, communicate more effectively with healthcare professionals, and make more informed decisions about their health [28].

An important aspect is the participation rates in preventive programs. While coverage of some programs, such as breast and cervical cancer prevention, is increasing, rates in others, especially influenza vaccination, remain low and unstable. This may be related to insufficient public awareness, misconceptions, misinformation or distrust in the healthcare system. Previous studies have shown that lower HL is associated with lower use of preventive healthcare services and poorer understanding of the benefits and risks of vaccination [29]. Therefore, HL becomes one of the most important factors determining residents’ decisions to participate in preventive programs and choose health-promoting behaviors.

This study has certain limitations. First, secondary aggregated data were used, so it was not possible to assess individual-level associations between HL and health indicators. Second, HL was not directly measured, but interpreted based on the literature and indirect indicators. Therefore, the study does not allow causal inferences regarding the relationship between HL and the analyzed health indicators, and the findings should be interpreted as descriptive and contextual rather than explanatory. Third, possible methodological differences in data collection between periods could have affected the comparability of results. In addition, the analyzed indicators covered different reporting periods depending on data availability, which limits direct comparisons across indicators. Nevertheless, the study provides important insights into possible associations between health indicators and HL in Lithuania.

In addition, the analysis used indicators that were not age-standardized. Therefore, the observed trends in indicators may partly reflect not only the actual changes in the burden of disease, but also demographic changes in the Lithuanian population, in particular the aging of the population and the decline in the population during the period under analysis. This should be taken into account when interpreting long-term trends in indicators. An important limitation is that trends in changes in indicators over time were assessed in a descriptive manner, using visual analysis of time trend data. No formal statistical trend analysis was performed. Furthermore, the comparison of municipalities was based on a simplified categorization into more urbanized and less urbanized areas, which may not fully capture the diversity of socioeconomic conditions, healthcare accessibility, and health system characteristics across municipalities. Finally, data collected during the COVID-19 pandemic period (2020–2022) may have been influenced by disruptions in healthcare utilization, disease detection, preventive service delivery, and reporting practices, which should be considered when interpreting the findings.

The findings of this study also have practical implications for the organization of primary healthcare and chronic disease management. In particular, the observed burden of chronic diseases, regional differences in avoidable hospitalizations, and the importance of HL highlight the need to strengthen multidisciplinary primary healthcare services. The family doctor team plays a crucial role in primary healthcare, as it is usually the patient’s first point of contact with the health system and ensures early diagnosis of diseases, monitoring of chronic diseases and patient education. Integrated team care improves treatment coordination and can reduce the number of avoidable hospitalizations. The role of nurses in patient education, strengthening self-management of chronic diseases and shaping health-promoting behaviors is becoming particularly important, especially among patients with limited HL [21]. Research also suggests that interventions aimed at improving HL can have a positive impact on treatment adherence and chronic disease management outcomes [25]. In Lithuania, these activities could be further strengthened through multidisciplinary primary healthcare teams and the ongoing primary healthcare reforms aimed at improving accessibility, continuity, and patient-centered care.

The results of this study have important practical implications for health policy. In order to reduce regional disparities, improve chronic disease control, and reduce the number of avoidable hospitalizations, it is necessary not only to improve the accessibility and quality of healthcare services, but also to systematically strengthen the HL of the population. This could be implemented through community-based educational programs, patient-centered communication, health education in schools, and more active integration of HL interventions into primary healthcare services. These activities could be integrated into existing Lithuanian health policy initiatives, including the implementation of the National Health Strategy and ongoing strengthening of primary healthcare services, particularly in less urbanized municipalities where access to healthcare resources remains more limited.

## 5. Conclusions

The main findings of this study show that NCDs remain the dominant health problem in Lithuania. CVDs account for more than half of all deaths, while the registered prevalence of cardiovascular diseases and type 2 diabetes continues to increase. Regional differences in avoidable hospitalizations and uneven participation in preventive services indicate persistent disparities in healthcare utilization. These findings highlight the continuing burden of chronic diseases on the Lithuanian healthcare system and the need to strengthen primary healthcare services. The observed burden of chronic diseases should also be interpreted in the context of demographic aging and the high prevalence of lifestyle-related risk factors reported in previous studies.

These findings support the importance of considering health indicators in the context of HL. However, as HL was not directly measured, its relevance should be interpreted based on evidence from previous scientific literature rather than as a direct finding of this study.

The findings also emphasize the importance of multidisciplinary primary healthcare, including patient education and support for chronic disease self-management. In this context, nurses, as members of the primary healthcare team, play an important role in strengthening patients’ self-management skills and supporting health-promoting behaviors.

Future studies should directly assess HL using validated instruments and examine its association with health indicators and healthcare utilization in the Lithuanian population.

## Figures and Tables

**Table 1:** Data availability table.

Indicator	Time Period	Data Availability
Mortality	2024, 2019, 2015, and 2010	Selected reference years
Registered prevalence of CVD and type 2 diabetes mellitus	2014–2024	Annual data available
Avoidable causes of hospitalizations	2023	Latest available data
Residents’ assessment of their own health	2005, 2014, and 2019	Survey years
Population visits to family and specialist doctors	2014, and 2019	Survey years
Influenza vaccine vaccination for people aged 65+	2022, 2023, and 2024	Data available
Participation in breast cancer prevention	2022, 2023, and 2024	Data available
Participation in cervical cancer prevention	2014, and 2019	Data available

**Table 2:** Healthcare service utilization in Lithuania.

Indicator	Year	Male (%)	Female (%)	Total (%)
Population visits to family doctors	2014	66.5	80.9	74.4
	2019	66.4	79.6	73.6
Population visits to specialist doctors	2014	33.9	41.1	37.9
	2019	30.5	39.0	35.1

**Table 3:** Participation in preventive programs in Lithuania.

Program	Target Population	2014 (%)	2019 (%)	2022 (%)	2023 (%)	2024 (%)
Breast cancer screening (mammography)	Women aged 50–69 years	–	–	57.6	59.7	61.2
Cervical cancer screening	Women aged 25–59 years	–	–	–	60.3	74.0
Colorectal cancer screening	Adults aged 50–59 years	13.6	26.1	–	–	–
	Adults aged 60–69 years	20.7	36.7	–	–	–
	Adults aged 70–74 years	22.9	39.5	–	–	–
Influenza vaccination	Population aged ≥ 65 years			22.5	24.1	22.4

Notes. Data were not available for the respective reporting years. The presented percentages refer to participation in nationally funded preventive programs and influenza vaccination among the corresponding target populations defined by the national programs.

## Data Availability

The supporting data used in this study can be accessed from the following official Lithuanian resources: Lithuanian Institute of Hygiene Health statistics: https://www.hi.lt/sveikatos-statistika/ (accessed on 10 January 2026) [10]; Lithuanian Official Statistics Portal: https://osp.stat.gov.lt/ (accessed on 10 January 2026) [11]; Lithuanian Institute of Hygiene’s Report on Avoidable Hospitalization Rates in Lithuania (2024): https://sam.lrv.lt/public/canonical/1733755205/26544/I%C5%A1vengiam%C5%B3%20hospitalizacij%C5%B3%20rodikliai%20Lietuvoje.pdf (accessed on 10 January 2026) [12].

## Abbreviations

The following abbreviations are used in this manuscript:

CVD	Cardiovascular disease
EU	European Union
HL	Health literacy
NCDs	Non-communicable diseases
